# Stakeholder perspectives on managing the adolescent sleep crisis using a transdiagnostic self-management app for sleep disturbances: A qualitative follow-up study

**DOI:** 10.1177/13591045241285586

**Published:** 2024-09-13

**Authors:** Parky H Lau, Colleen E Carney

**Affiliations:** 1 Department of Psychology, 7984 Toronto Metropolitan University, Canada

**Keywords:** Sleep health, teen sleep, evidence-based intervention, reflexive thematic analysis

## Abstract

Sleep problems are diverse and pervasive among the adolescent population. Current sleep health interventions are ill-equipped to address the sleep crisis. We developed DOZE (Delivering Online Zzz’s with Empirical Support), which is a self-management evidence-based app for sleep disturbances. In an initial study, we found that DOZE was perceived as an acceptable and effective support for teen sleep. In a qualitative follow-up study, we engaged with students and other stakeholders to understand their experiences with implementing, disseminating, and using DOZE. The study employed a combination of qualitative surveys and semi-structured interviews to students (*n* = 21) and stakeholders (teachers, social workers, and researchers; *n* = 5), respectively. Reflexive thematic analysis was used to identify themes related to experiences implementing and engaging with the app. Students reported increased sleep regularity and sleep duration after using DOZE. Facilitators included greater integration of the app with school curriculum, timing of implementation, and researcher involvement in supporting knowledge dissemination and engagement. Barriers included worries about phone use at night and normalized poor sleep patterns among adolescents. There is need to identify ways to support implementation and engagement in different communities. Researchers continue to engage with stakeholders to support timely access to sleep health interventions for adolescents.

## Plain language summary

High school students struggle with many sleep problems, which then causes problems in their health and ability to do well in school. However, regular solutions to improving sleep in teens ask them to go bed early, which is not helpful because teens tend to be 'night-owls'. To tackle this problem, we developed a teen sleep app based on sleep science (DOZE) that is free and accessible for high school students to use. We found that students found this app to be enjoyable to use and helpful in supporting sleep health. Our next step was to use survey questions and interviews to learn more from teachers, social workers, and students about their experience getting DOZE into the school and experiences using the app. We wanted to know what individuals liked about the app, what made it easier/harder to bring DOZE into the school, and how we can continue to support sleep health in adolescents. We found that students were more likely to use DOZE when the app was placed within the school curriculum (e.g., physical education courses) and when the researchers were more involved - such as by giving talks. On the other hand, some individuals were worried about the use of phones during the night time and some students figured that poor sleep is a normal part of the high school experience. Overall, individuals generally found DOZE to be helpful and social workers noted that they will continue to suggest DOZE for students that struggle with poor sleep. In terms of policy recommendations, we need to continue thinking about ways to support sleep health, such as by delaying school start times to give adolescents more time to sleep. We continue to work with teachers and social workers in Canadian high schools to understand how to support teen sleep.

High school adolescent struggle with sleep disturbances. Epidemiological studies suggest that chronic sleep deprivation is a worldwide epidemic, with approximately 50% of adolescents not meeting the American Academy of Sleep Medicine guidelines for recommended sleep duration (Gradisar et al., 2011; Paruthi et al., 2016). Biological reasons for this shortened sleep include pubertal maturations shifting circadian rhythms towards a more delayed phase (i.e., eveningness; Roenneberg et al., 2004) and slower build-up of homeostatic pressure for sleep (Jenni et al., 2005). Moreover, other psychosocial factors such as social commitments and electronic use that can further exacerbate delayed schedules in adolescence (Hysing, Haugland, et al., 2015; Pea et al., 2012). In the morning, early school start times then conspire with these aforementioned biopsychosocial factors to prohibit sufficient sleep opportunity (for a review, see Kirby et al., 2011).

The mismatch between psychosocial pressures and biological maturations can also engender other sleep problems. For example, symptoms of insomnia, such as difficulties falling asleep, can result because adolescents may attempt to go to bed before their body’s natural preferred timing for sleep. Moreover, ‘jet lag’ symptoms can arise because of circadian misalignment from irregular sleep-wake patterns (i.e., going to bed and waking up later on weekends; Foster et al., 2013). These myriad sleep disturbances cascade into health-related difficulties in various domains (e.g., Matthews & Pantesco, 2016; Palmer et al., 2018; Zhang et al., 2018), in addition to deficits in cognitive and academic performance (Beebe, 2012; Hysing, Pallesen, et al., 2015).

Adolescent sleep is consequently widely recognized as a public health crisis (Owens et al., 2014). There exist various effective non-pharmacological treatment options for sleep problems (Bourchtein et al., 2020). Unfortunately, traversing the bridge between the excellent work in behavioural sleep medicine and the adolescent population for whom this work can serve is a challenging clinical quandary. There are limitations to access at the individual and systems level. Behavioural sleep medicine – though indeed effective – is lacking in scalability because mental health professionals who are trained to provide these services are low and most training opportunities are limited (e.g., Manber et al., 2012; Thomas et al., 2016). Moreover, adolescents may be largely unaware of treatment options or do not have the resources to access these services (for a scoping review, see Garney et al., 2021). In terms of symptom presentation, adolescents can experience myriad sleep problems across a spectrum of severity, which requires a flexible and transdiagnostic intervention that can be modified based on user needs and preferences. Therefore, protocol-driven or disorder-specific sleep treatments may not be commensurate with the requirements needed to support fulsome sleep health in adolescents.

The fact is that available sleep health interventions tend to be too specific or resource inefficient to provide adequate support to the myriad and pervasive sleep disturbances in adolescents. To address these limitations, we spearheaded DOZE (Delivering Online Zzz’s with Empirical Support), which is a transdiagnostic self-management app for sleep disturbances in adolescents and young adults. DOZE uses daily sleep diaries to provide data-driven recommendations based on the users’ goals. The flexible and personalizable nature of this app – developed in consultation with adolescents and young adults and their feedback – has been found to be an efficacious and credible option (for a study on design, feasibility, and efficacy of DOZE, see Carmona et al., 2021).

More recently, we collaborated with stakeholders at Canadian high schools to implement and disseminate DOZE within the school system with minimal researcher supervision and incentive to participate. This project is currently being considered for publication elsewhere. The purpose was to evaluate whether DOZE could reliably be employed into the community to high acceptability and effectiveness. Students completed four weeks of sleep diaries where they were able to set goals and receive feedback after the initial two weeks of monitoring. Results found that students perceived DOZE to be easy to use, understandable, and time-efficient. From an effectiveness lens, the app was helpful in regularizing bed and rise times, increased sleep duration, and reduced perceived symptoms of insomnia, suggesting that DOZE could be one solution towards broad-based access to evidence-informed sleep healthcare.

The acute stage of trialing this intervention appears to be a success. However, we wished to follow-up with stakeholders and engage with the community as part of a collaborative effort in understanding next steps to support sleep health in adolescents. Given the community-based nature of this work, there is a need to dovetail the expertise of researchers with stakeholders in the community that have lived experience in supporting the implementation, dissemination, and use of DOZE. This type of community-based participatory research creates trust and collaboration, honoring the knowledge and expertise of all parties and ensuring that the conclusions and recommendations drawn are consistent with the needs of the community (e.g., Collins et al., 2018). The present study is a qualitative investigation with those who used DOZE (i.e., students) and stakeholders who supported its implementation (i.e., teachers, social workers, researchers) to understand several key domains of inquiry: 1) perceptions and experience with using DOZE app, 2) barriers and facilitators to implementation and engagement, 3) perceived benefits and limitations of DOZE in supporting sleep health, 4) outstanding areas of sleep concern, and 5) further strategies and recommendations to address the adolescent sleep crisis. It is our hope that this qualitative endeavour can lead to the co-construction of knowledge that further bridges the scientific practice of behavioural sleep medicine and the community.

## Methods

### Study participants

Study participants included two primary groups. One group included stakeholders that supported implementation and dissemination of DOZE. These were individuals that responded to the initial outreach for collaboration with the investigators. The roles of these individuals at the school were varied, with a combination of teachers, social workers, and research leads. The second group included Canadian high school students between the ages of 11 to 17 who participated and used DOZE. The students filled out baseline questionnaires and then used DOZE for four weeks (completing daily sleep diaries). After the initial two weeks, students were able to set goals and received feedback on how to improve their sleep (e.g., falling asleep quicker, having a more consistent schedule, etc.). Students then completed endpoint questionnaires after the four-week period, which included open-ended qualitative survey questions.

### Qualitative interviews and surveys

There were two methods of qualitative data collection used in this study: interviews and survey methods. For stakeholders who supported implementation and dissemination, a semistructured interview approach was used as an inter-change of information between two individuals about themes of mutual interest (Kvale, 1996). This semistructured nature honours the fact that each individual played different roles in supporting the implementation of DOZE and may therefore have greater depth of knowledge in certain domains of inquiry. Consequently, this flexible approach allows the researcher and participation to organically engage in the collaborative co-creation of knowledge (DiCicco-Bloom & Crabtree, 2006).

Students completed qualitative surveys asking questions about their experience with DOZE and how the school can best continue sleep needs. The survey method, rather than interview, was used to encourage disclosure in a safe setting for a vulnerable population. Moreover, the higher sample size meant that a survey method would permit a greater response sample, while reducing overall participant and investigator burden that may result from the use of an interview approach (Braun et al., 2021). See Table 1 for a list of questions used to guide the interviews.

**Table 1.** List of interview questions for stakeholders.

Implementation
1. What was your experience with the process of implementing DOZE?
2. What made it easier to implement the app into the existing school structure? What made it harder?
3. What would be helpful in the future to support implementation?
Engagement
1. Were there any barriers that you noticed that affected student engagement with DOZE?
2. Were there any facilitators (i.e., what made engagement easier)?
3. How might we increase student interest/engagement with the app in the future?
Effectiveness
1. Did you notice any improvements in student sleep or other outcomes (such as mood, energy, productivity) after using the app?
2. What components did you think was effective in helping students?
3. What barriers do you think affected the app’s effectiveness? How might we improve this issue?
Continued supports
1. What additional sleep challenges do you think students are still experiencing?
2. How might we continue to help students sleep well during high school?
3. How is DOZE limited to address these ongoing issues?
*Survey questions for students
How do you feel about your sleep after using DOZE app?
What did you like about DOZE app?
What did you not like about DOZE app?
What else can the school do to help improve your sleep?

*Note.* The interviews were semi-structured and therefore used as guides. Questions or modified or removed depending on stakeholder’s expertise with supporting DOZE’s implementation and engagement.

### Procedures

The investigator reached out to stakeholders via email correspondence after the completion of the four weeks of initial study. The email provided information on a qualitative interview study with the stakeholder to discuss their experience with DOZE and identify ways to further support adolescent sleep health in the community. To reduce coercion, it was emphasized that their engagement in the study would not impact their relationship with the investigator, the university, or the ability to continue using the free sleep health app. Interviews were conducted using videoconferencing. Interviews were transcribed on Otter.ai and uploaded onto a secure university file drive. These files were de-identified and assigned an ID. Students completed qualitative surveys as part of the endpoint questionnaires. Interview participants received a US$25.00 gift card as an honorarium for their participation in the study. There were no incentives for students as this was part of a larger study that looked at whether students would use the sleep app with minimal study incentive. All participants provided their informed consent and the study received ethics approval from the university.

### Qualitative data analysis

Reflexive thematic analysis (TA) was utilized as the primary approach for handling and interpreting qualitative data for this paper (Clarke & Braun, 2017). Thematic analysis is a method for identifying, analyzing, interpreting, and reporting patterns of meaning (i.e., themes) within qualitative data. TA can be applied across a range of theoretical frameworks and paradigms, and is flexible in working with research questions, sample sizes, and data collection methods (Clarke & Braun, 2017). Braun et al. (2018) offered a tripartite typology of TA: cluster and call coding coding reliability, codebook, and reflexive approaches. (Post-)positive coding approaches, known as ‘small q’ qualitative methodology, tend to leverage more structured approaches to coding and theme development (Boyatzis, 1998; Guest et al., 2012). These approaches, which include additional coders to increase interrater reliability, are sometimes seen as more empirically ‘reliable’ through limiting researcher bias (see Braun et al., 2016). In so doing, these researchers position TA within a (post-)positivist and realist (‘factist’) research framework, where it is assumed that ‘truth’ and demarcated meaning can be inherently found through data (Ho et al., 2017).

On the other hand, we offer a purely qualitative framework that is theoretically flexible and argue for the use of a more interpretative and reflexive approach by creating meaning through the researchers’ lens rather than assuming an objective truth. Specifically, Braun and Clarke (2019) contend that themes are actively generated rather than passively discovered, highlighting the researcher as an important component in the intersection of data, analytic process, and subjectivity. As a result, we intended our approach to TA to reflect a fully qualitative approach (‘big Q’) as creative, reflexive and subjective, with researcher subjectivity perceived as essential in meaning-making and interpretation rather than a detriment to scientific inquiry (see Gough & Madill, 2012). In this way, the researcher’s reflexive engagement with the data forms the crux of knowledge production and interpretation.

Thus, the use of reflexive TA in this manuscript is meant to leverage the perspective and expertise of the investigator as a student, researcher, clinician, and educator of behavioural sleep medicine. It is important to acknowledge this lens so readers are aware of the position that the investigator takes as part of recursive analysis and interpretation regarding the generative component of TA. The qualitative data obtained explores private high school students’ experiences in relation to their perceived sleep and factors that act as facilitators or barriers to sleep health. The six phases outlined by the inaugural paper by Braun & Clarke (2006) were used to support adherence to the TA approach: 1) familiarization with data 2) generating initial codes 3) searching for themes 4) reviewing themes 5) defining and naming themes and 6) producing the report.

In terms of specific steps, the surveys and interviews (particularly relevant in the third part of this project) were transcribed verbatim and anonymized with a random ID. The data was then read several times to increase familiarity with the data. Data analysis had a dual focus on immersion followed by distancing in order to properly reflect on burgeoning codes and themes (Braun & Clarke, 2022). Initial codes were then generated and then refined over time through continual reflections and engagement with the data. For example, after coding each individual line, the researcher then reflexively engaged with patterns and codes within and across transcripts and qualitative survey responses. Given that reflexivity is a key aspect of thematic analysis, the researcher conducted the work alone to engage in a deeper personal reflection and support an authentic and nuanced analysis based on his perspectives and biases. Unlike coding reliability approaches, we believe that coding is recognised as an inherently subjective process and requires a reflexive researcher to evaluate and reflect on how their assumptions and biases shape coding (Braun & Clarke, 2021). The researcher is well-positioned to ensure quality and depth of analysis as someone with a developed background in behavioural sleep science, research, and practice.

## Results

### Participant characteristics

Respondents included 21 students and five stakeholders (three social workers, one teacher, and one researcher). Besides profession, stakeholder demographics were not obtained. One stakeholder did not respond to the researcher’s call to participate. Students (mean age = 14.8, SD = .79) were primarily male (94.7%) from European descent (42.1%). See Table 2 for a breakdown of participant demographics.

**Table 2.** Participant Characteristics for students and stakeholders.

Variable	Mean (SD) or Count (%)
Students (*n* = 19)	
Age	14.8 (0.79)
Sex	
Female	1 (5.3%)
Male	18 (94.7%)
Ethnicity	
Caribbean	1 (5.3%)
East/Southeast Asian	5 (26.3%)
European	8 (42.1%)
Other/Prefer Not to Answer	5 (26.3%)
School grade	
Grade 9	9 (47.4%)
Grade 10	6 (31.6%)
Grade 11	4 (21.1%)
Stakeholders (*n* = 5)	
Role	
Social worker	3 (60.0%)
Teacher	1 (20.0%)
Head of Innovation	1 (20.0%)

### Perceived effectiveness and acceptability of DOZE

#### Positive experiences with DOZE in supporting sleep health

Students generally reported positive experiences with DOZE. Specifically, students noted that the app was effective in increasing total sleep time and regularizing their schedule. One student stated that the tracking of their sleep naturally supported their desire to improve their sleep.

“I think my sleep was on an upwards trajectory. Knowing you're tracking it, subconsciously you want to make your sleeping hours as high as possible and regulate them.” [Student 94201]

#### Daily diaries increases awareness of sleep patterns

A social worker reported that in her counselling sessions, students found that DOZE was particularly helpful in noticing patterns for when sleep was more disrupted. The students were able to connect these stressors with sleep disturbances, which increased awareness of causes of poor sleep.

“For [students] in particular, it was helpful to be able to have a look back at times where there was uninterrupted sleep versus times where there were, and just want to be able to look at a distance and say, “What were the things that impacted sleep for me?” [Stakeholder 1, Social Worker]

#### Neutral Reactions towards DOZE

Some students had a more neutral response to DOZE indicating that the tracking was neither beneficial nor harmful to their sleep. They indicated that their sleep was either related to causes that DOZE does not adequately handle, such as extent of homework or that their sleep was “about as good as it can be – my sleep did not improve.” [Student 40137]

“I do not believe the app helped because my schedule mostly is troubled by external factors like homework, school, work, and extra-curriculars. It is not lack of goal planning, merely a lack of hours in the day.” [Student 28492]

#### DOZE as clear, easy to use, and time efficient

In terms of acceptability, students perceived DOZE as generally acceptable, being easy to use and time-efficient. Specifically, many students identified the user interface of DOZE to be easy to understand and clear. They also noted the user interface was “aesthetically pleasing” [Student 59690].

### Facilitators and barriers of implementation and engagement

#### Different levels of integration at the school level

One stakeholder noted that implementation is highest when the research is co-designed with the school or is fully integrated into subject curriculum. This was contrasted with lower levels of integration, such as when DOZE was simply advertised as part of a general newsletter.

“The highest level of engagement is when an external researcher co-designs their research initiative. That's some kind of classroom intervention that integrates with the curriculum of that teacher, subject, area, and grade.” [Stakeholder 5, Head of Innovation].

#### Researcher involvement in supporting implementation

One social worker identified that the researcher involvement was helpful in supporting implementation by bringing knowledge and clarity in the process. They noted that engagement at the administrative level is facilitated when the researcher supports this process given typical work demands.

“I really appreciated the clarity with which you explained the program and the app, and your willingness to create a promotional video for students. That made it really easy on our end to like, engage with you.” [Stakeholder 3, Social Worker]

Another stakeholder also discussed the utility of facilitating the process for teachers is helpful to reduce burden and increase buy-in to the project.

“I mean, that's, that's actually probably like, really ideal too, because in a sense, you're not really taking up a lot of teachers time, right? And if the teachers already teaching about sleep, then this supplements what they're already doing.” [Stakeholder 5, Head of Innovation]

#### Student priorities as barriers to sleep health

A commonly discussed barrier to student engagement with improving their sleep was competing demands. Sleep was seen as less important compared to other priorities, such as social, academic workload, and extracurricular activities. Consequently, sleep is often sacrificed to support commitment in these other activities.

“Students are feeling like they need to make choices and prioritize academics and co-curricular pursuits. And sleep quite often is low or feels like that can be the one thing that's let go of.” [Stakeholder 3, Social Worker]

#### Timing of implementation

A teacher identified that timing of dissemination is an important factor, as some periods may be more conducive to having adequate time and resources to integrate DOZE. For example, Fall tends to be a more challenging time for students because of university applications. On the other hand, integrating the app during daylight time savings or a mental well-being week may be a more opportune moment.

“I know like once November hits, it's a busy time for university applicants…But like, if we added it in into one of our mental well-being weeks where we can promote it more as part of something else, not just a one-off helpful solution” [Stakeholder 4, Teacher]

#### The Role of web-based apps in engagement

One stakeholder identified that the accessibility of a web-based app was particularly important for “meeting students where they are at.” On the other hand, they noted that the phone use may be contributing worries that this app might hinder sleep: “Conversely, we’re often talking with young people and parents about not having any devices in their room. So that that is maybe one potential tension?” [Stakeholder 1, Social Worker].

### The role of family in supporting engagement

One social worker identified that it would be helpful to engage with parents about sleep and DOZE to support a community-based approach.

“I wonder like, if there was like a parent piece to say, hey, this can be really useful. Because I find that when there's that home connection, we often get the best results.” [Stakeholder 1, Social Worker]

### Continued areas of sleep concern in adolescents

#### Psychosocial factors as disruptors of sleep

Both students and other stakeholders identified psychosocial factors as ongoing issues that act as barriers to sleep health. For example, a combination of homework, early school start times, and evening co-curriculars were seen as common disruptors.

“So we end our day at around 2:40 pm. They'll stick around and do some school-related things, clubs and teams, and then they typically will go home and maybe play a nine o'clock hockey game or stay at school till 9:30 pm practicing for the musical.” (Stakeholder 1, Social Worker]

Another stakeholder identified nighttime phone and computer use affecting student’s sleep opportunity.

“The main problem is phone technology…so students are on their phones on their laptops till 1 or 2 o'clock in the morning” [Stakeholder 4, Teacher]

#### Poor sleep as normalized in students

There was a prevailing belief that poor sleep is simply the norm in high school, which may contribute to maintenance of poor sleep habits.

“[Poor sleep] is a normalized kind of condition that you come to school tired and that you kind of make up for it at the weekend.” (Stakeholder 2, Social Worker].

### Further strategies to support sleep health in adolescents

#### Reflections on policy recommendations

Many students identified starting school at a later time and less homework as strategies to support sleep health. Stakeholders also identified that past changes in school start times have been helpful to support overall health and wellbeing.

“So actually, our start time used to be 8:00 am years ago. And it's up to 8:45 am. I think that 45 minutes has helped a lot” [Stakeholder 4, Teacher]

#### Continued availability of DOZE in high schools

There was discussion on the continued availability DOZE app to continue supporting adolescent sleep health, especially those who want additional help with sleep.

“Nice to have an app that's continued to be free and that students can use if and when they feel like the problem is big enough that they want some sort of more formal intervention.” (Stakeholder 2, Social Worker)

One social worker identified that they would introduce DOZE to a class and compare this with other apps (e.g., Calm) as part of focus groups.

#### Future researcher involvement in community engagement

There was discussion on opportunities in the future for researcher involvement in the form of psychoeducational talks to discuss the benefits of sleep with students, teachers, and parents.

“I think when you came in, you gave us some information that they didn't know and it was a really good presentation. So I would certainly love to see you come back during another opportunity, like a mental health week or you know” (Stakeholder 2, Social Worker).

See Table 3 for a Summary of domains and themes.

**Table 3.** Domain and Themes.

Domain	Theme
Perceived effectiveness and acceptability of DOZE	Positive experiences with DOZE in supporting sleep health
	Daily diaries increase awareness of sleep patterns
	Neutral reactions towards DOZE
	DOZE as clear, easy to use, and time efficient
Facilitators and barriers of implementation and engagement	Different levels of integration at the school level
	Researcher involvement in supporting implementation
	Student priorities as barriers to sleep health
	Timing of implementation
	The role of web-based apps in engagement
	The role of family in supporting engagement
Continued areas of sleep concern in adolescents	Psychosocial factors as disruptors of sleep
	Poor sleep as normalized in students
Further strategies to support sleep health in adolescents	Reflections on policy recommendations
	Continued availability of DOZE in high schools
	Future researcher involvement in community engagement

## Discussion

In general, stakeholders and students saw DOZE as a useful and effective tool for supporting sleep health. Students reported that the app was helpful in increasing sleep time, regularizing timing of bed and rise schedules, and the tracking element of DOZE allowed them to able to notice patterns in their sleep. Although the absence of evidence is not necessarily an absence of phenomena, the fact that there were no discussion of adverse events related to DOZE’s impact on well-being is also important to note. This suggests that the app maintains the ethical principle of non-maleficence in reasonable fashion even if it does not improve sleep in every student. The lack of adverse events is cardinal to DOZE as it necessarily requires less oversight and clinician involvement. The reason is that DOZE is meant to be an accessible and scalable way to increase behavioural sleep medicine access in the community.

Students and stakeholders both identified several facilitators of engagement. Specifically, student indicated that DOZE was easy to use, time-efficient, with a simple and intuitive interface. At the school level, stakeholders identified that the researcher’s clarity about sharing information about DOZE and willingness to engage with the community through talks and promotional videos was helpful to support engagement. They further identified that different levels of integration significant impacts engagement, with co-integration of DOZE with the curriculum leading to the highest levels of engagement.

On the other hand, one significant barrier to engagement that several stakeholders reported was the impact of competing demands and priorities that limited students desire to focus on sleep. Although students are likely aware of the benefits of sleep, it would be helpful to make this relationship between sleep and performance explicit through psychoeducational talks. This may provide insight into the paradoxical nature of prioritizing time to sleep to facilitate other values, such as academics and sports (Hershner, 2020; Potter et al., 2020). In order to address other possible barriers, consulting with schools to decide on proper timing of dissemination as well as further community engagement to 1) emphasize the value of sleep, 2) dispel beliefs about the need for nighttime phone use, 3) involve parents in this process.

### Limitations of the present study

The current study was conducted in private schools, which may be unique in terms of their specific infrastructure and bandwidth in supporting and integrating research initiatives. There is further need to engage in reach out to public schools to evaluate engagement and obtain data to determine how to best support different communities. Moreover, given the community-based nature of the research, it would also be beneficial to outreach to parents to obtain a more fulsome approach to supporting sleep needs. Finally, the co-construction of this type of integrated research with the community needs to be ongoing. This present discussion represents reflections after four-week period of using DOZE. Continuing this work with schools will be helpful to identify challenges associated with maintenance in engagement with DOZE.

### Summary and next steps

Overall, the current qualitative investigation with stakeholders and students identified DOZE as an acceptable and effective option to support sleep health. The research also identified potential facilitators and barriers to implementation in engagement. Specifically, a focus on greater integration of DOZE into existing curricula, considerations of timing of implementation, and researcher involvement through community psychoeducation talks were discussed.

Beyond these considerations, there is need to further need to discuss how we as sleep scientists can construct services that continually support the myriad and varied community sleep needs over time. DOZE is not meant to be a panacea to the adolescent sleep health crisis. As noted, there are continued sleep challenges that require different solutions to properly address. Consequently, there is still a need to position DOZE within a larger network of sleep health supports, services, and policy recommendations.

One important reason for this nuance is that sleep health needs and causes of sleep problems are different depending on the student. For example, some students noted that their sleep was already adequate or that the maintaining factors of sleep problems were less likely to be addressed using DOZE strategies (e.g., challenges with school start times and co-curricular activities, amount of assigned homework). Therefore, these challenges may require a negotiation surrounding policy recommendations about homework, timing of co-curriculars, or delaying school start times. Moreover, a stepped care model for interventions could also be helpful. Students with adequate sleep health may require lower intensity psychoeducation or self-management sleep apps like DOZE, whereas students with greater difficulties with sleep may be triaged to higher intensity clinician-supported sleep treatments (e.g., transdiagnostic sleep interventions; Harvey, 2016). This can ensure that we meet students where they are in terms of their sleep needs and hierarchy of priorities.

We are currently continuing to engage with stakeholders to position this app within a wider framework of strategies to support adolescent sleep health. In the interviews, some social workers have said that they will continue to suggest DOZE to students reporting sleep issues, which remains free and accessible to students. One social worker is also planning to recommend DOZE in an email to parents during daylight saving time. Another teacher is planning to conduct a focus group with her students to compare DOZE with other apps (e.g., Calm app). The researcher continues to be a liaison with stakeholders to support future engagement through psychoeducational talks and supporting the use of DOZE.
